# A Novel Resveratrol-Arsenic Trioxide Combination Treatment Synergistically Induces Apoptosis of Adriamycin-Selected Drug-Resistant Leukemia K562 Cells

**DOI:** 10.7150/jca.34506

**Published:** 2019-08-29

**Authors:** Jing Chen, Baoying Tian, Cunmin Zhou, Jingjing Sun, Li Lin, Shucheng Jin, Quanrui Liu, Siyu Fu, Lian Liu, Hang Liu, Zhewen Zhang, Caili Li, Hulai Wei

**Affiliations:** 1 Key Laboratory of Preclinical Study for New Drugs of Gansu Province, School of Basic Medical Sciences, Lanzhou University, Lanzhou, Gansu, 730000; 2 Hanzhong vocational and technical college, Hanzhong, Shanxi, 723000; 3 School of Medicine of Northwest University for Nationalities, Lanzhou, Gansu 730030, P.R. China

**Keywords:** leukemia, multidrug resistance, arsenic trioxide, resveratrol, apoptosis

## Abstract

Leukemia cells can develop resistance to apoptosis induced by chemotherapeutic agents. Concomitant multidrug resistance of cells remains the greatest clinical obstacle in the effective treatment of blood and solid tumors. Natural products have been identified that possess the capacity to modulate chemotherapeutic resistance and induce apopotosis. In this study, we generated adriamycin-resistant K562 leukemia (K562/RA) cells and compared the responses of sensitive and resistant leukemia cells to the natural products arsenic trioxide (ATO) and resveratrol (Rsv), with a view to determining whether Rsv potentiates the sensitivity of drug-resistant cells to ATO-induced apoptosis and the associated molecular mechanisms. Our results showed that resistance of K562/RA cells induced by adriamycin treatment was significantly higher (115.81-fold) than that of parental K562 cells. Simultaneously, K562/RA cells were cross-resistant to multiple agents, with the exception of ATO. Rsv enhanced the sensitivity of K562/RA cells to ATO and reduced the required dose of ATO as well as associated adverse reactions by promoting the proliferation inhibitory and apoptosis-inducing effects of ATO, which may be associated with reduced expression of the drug resistance genes mdr1/P-gp, mrp1/MRP1 and bcrp/BCRP, as well as the apoptotic inhibitory genes bcl-2, NF-κB and P53, and conversely, activation of caspase-3. Our collective findings indicate that ATO and Rsv synergistically enhance the sensitivity of drug-resistant leukemia cells to apoptosis.

## Introduction

Long-term or repeated use of specific chemotherapy drugs can induce multidrug resistance of leukemia cells, even at therapeutic doses 1-3. Reduced apoptosis-inducing activity of chemotherapy drugs is an important mechanism contributing to the development of multidrug resistance (MDR) in leukemia cells 4-6. Identification of novel drugs that have no cross-resistance with conventional chemotherapy drugs and development of agents that enhance the apoptosis-inducing effect of currently available drugs are potentially effective measures to overcome MDR of leukemia. Arsenic trioxide (ATO) has good efficacy for many leukemia and solid tumor types with fewer side-effects than other agents 7-11. In acute promyelocytic leukemia patients who achieve remission after retinoic acid treatment, combination therapy with ATO has been shown to consolidate the efficacy of retinoic acid. ATO is thus considered the 'gold partner' of retinoic acid 12,13. Although results obtained with ATO provide an optimistic perspective in the management of leukemia, the compound exerts cytotoxicity, which causes damage to human organs. The majority of patients treated with ATO (~75%) experience grade 1 or 2 liver dysfunction 12-14. In addition, a number of patients in remission after treatment with a combination of retinoic acid and ATO experience relapse. These patients often carry the missense mutations A216V, L218P, A216T, S214L, L217F and S220G in the promyelocytic leukemia protein-retinoic acid receptor α (PML/RARα) domain, which induce arsenic resistance, and consequently, a significant increase in mortality 12-14. *In vitro* studies have confirmed a marked apoptosis-inducing effect of ATO on leukemia cells, especially those that are drug-resistant. However, divalent, trivalent and pentavalent arsenic intermediates have been detected in saliva, urine and blood of ATO-treated patients in clinical trials. The toxic effects of accumulated arsenic on the human liver, kidney and cardiovascular system cannot be overlooked 14-16.

Resveratrol (Rsv) is a natural antioxidant that not only has anti-viral and immune modulation properties but also prevents mutation and tumor development 17,19. Rsv is reported to significantly reduce the malondialdehyde and NO contents in rat serum, liver, spleen, lung and brain induced by ATO, suggestive of effective antiperoxidase activity 20,21. Rsv has been shown to enhance apoptosis of HeLa, MCF-7 and NB40 cells induced by ATO, both *in vitro* and *in vivo*. Moreover, Rsv protects normal cells against ATO toxicity by promoting apoptosis in tumor cells, although the precise mechanisms are currently unclear 22. In this study, we generated drug resistance in K562 leukemia cells using adriamycin (ADM) and compared the responses of sensitive and resistant cell groups to ATO and Rsv. Additionally, the effects of combined treatment with ATO and Rsv on drug-resistant leukemia cells and the associated molecular mechanisms were investigated.

## Materials and Methods

### Reagents

SYBR Premix Ex Taq (RR820A) and Prime Script RT reagents (RR036A) were obtained from Takara Bio (Otsu, Japan). P-gp (mdr1), MRP1 (mpr1), BCRP (bcrp) and β-actin primers were additionally synthesized by Takara. Mouse anti-β-actin (3598.100, BioVision, Milpitas, CA, USA), rabbit anti-BCL-2 (12789-1-AP; Proteintec, Chicago, USA), rabbit anti-BAX (50599-2-lg; Proteintec), mouse anti-P53 (BM0101), rabbit anti-NF-κB (PB0161), rabbit anti-P-gp (BA1351‑2), anti-BCRP (BA2307‑2), anti-MRP1 (BA0567) (all from Boster, Wuhan, China), rabbit anti-cleaved caspase-3 (YM3431; ImmunoWay, Plano, TX, USA) and P-gp antibodies (MRK16, Mc-012; Kamiya Biomedical, Seattle WA, USA) were used. The chemotherapeutic drug ADM was purchased from Shenzhen Wanle Pharmaceutical Co. (Shenzhen, China) and ATO and Rsv acquired from Sigma-Aldrich (Merck KGaA, Darmstadt, Germany).

### Cell culture and incubation

Human leukemia K562 cells were purchased from American Type Culture Collection (ATCC, Manassas, USA). Cells were maintained in RPMI 1640 medium (Gibco; Thermo Fisher Scientific, Inc. Waltham, MA, USA) supplemented with 10% fetal calf serum (Hyclone; GE Healthcare Life Sciences, Logan, UT, USA) and cultivated at 37°C in a 5% CO_2_ incubator.

### Generation of ADM-resistant cells

ADM resistance in human leukemia K562 cells was induced by long-term exposure to continuous stepwise increments of ADM. Cells were cryopreserved and recovered after 2 to 3 months of induction to prevent mutations until they were stably able to tolerate 16 μM ADM. This procedure was used to generate a drug-resistant leukemia subline, designated K562/RA.

### In vitro drug sensitivity analysis

A total of 0.8×10^5^ target cells/ml were plated in 96-well plates and cultured at 37°C with the specified concentrations of chemotherapeutic agents for 24-72 h. Absorbance was quantified using a Powerwave X plate reader (Omega Bio-Tek, Inc., Norcross, GA, USA). The MTT assay (in which each well was incubated with 5 mg/ml MTT for 4 h at 37°C, followed by overnight incubation with 100 µl 10% SDS at 37°C) was employed for determination of the half-maximal inhibitory concentration (IC_50_) values for ADM and cytotoxicity assays.

### Morphological features of apoptotic cells

Target cells were collected and stained with Giemsa-Wright stain (Solarbio, Beijing, China) according to the manufacturer's instructions. Morphologic changes were analyzed under an AX80 optical microscope (Olympus, Tokyo, Japan).

### Flow cytometric analysis of caspase-3 activity and apoptosis

For the caspase-3 activity assay, cells were collected and labeled with FITC-DEVD-FMK (BioVision, Milpitas, CA, USA) for 30 min, which irreversibly binds to activated caspase-3. Caspase-3 activity was directly determined using an Epics XL-4 flow cytometer (Beckman-Coulter, Brea, CA, USA).

### Real-time quantitative RT-PCR

Total cellular RNA was extracted using a TRIzol kit (Invitrogen; Thermo Fisher Scientific, Inc. Waltham, MA, USA) and cDNA was obtained by PrimeScript^TM^ RT reagent Kit with gDNA Eraser (RR047A, Takara Bio, Otsu, Japan). Amplification was performed with the following primers: MRP1, forward: 5′TGCAGAAGGCGGGGAGAACCTC3′, reverse: 5′GTCGTCCGTTTCCAGGTCCACG3′; P-gp, forward:5′CCCATCATTGCAATAGCAGG3′, reverse: 5′GTTCAAACTTCTGCTCCTGA3′; BCRP2, forward: 5′GCTGCAAGGAAAGATCCAAGT 3′, reverse: 5′TAGTTGTTGCAAGCCGAAGAG 3′, β-actin, forward: 5′-TGCTCCTCCTGAGCGCAAGTA-3′, reverse: 5′-CCACATCTGCTGGAAGGTGGA-3′. The PCR conditions included initial denaturation at 95˚C for 10 sec, 40 cycles of denaturation at 95˚C for 5 sec and annealing at 60˚C for 30 sec. Reactions were conducted using a Rotor-Gene 3000 quantitative PCR amplifier (CobetteRes. Inc, Sydney, Australia). The relative mRNA levels were calculated by comparison to that of β-actin via the 2^‑ΔΔCt^ method 23.

### Western blot analysis

Following lysis of target cells lysed using a radioimmunoprecipitation assay protein extraction reagent (P0013B; Beyotime Inc. Shanghai, China), proteins were fractionated via SDS-PAGE and transferred onto PVDF membranes (EMD Millipore, Billerica, MA, USA). Next, membranes were probed with primary antibodies and anti-β-actin antibody (Mouse mAb; BioVision, Milpitas, CA, USA), followed by IRDye800CW‑conjugated goat anti‑mouse secondary antibodies (926‑32210, dilution, 1:10,000, LI‑COR Biosciences, Lincoln, NE, USA) or IRDye680DX‑conjugated secondary antibodies (goat anti‑rabbit, 926‑32221, 1:10,000, LI‑COR Biosciences). Antibody-coated protein bands in immunoblots were quantitated and visualized using an Odyssey double-color infrared-laser imaging system (Odyssey v1.2 software; LI-COR Biosciences).

### Statistical analysis

All data are presented as means ± standard deviation. Multiple comparisons among the three groups were performed using one-way analysis of variance (ANOVA). Intragroup comparisons were conducted using the paired Student's t test. Error bars represent standard deviation, and *P*<0.05 indicates statistically significant differences between groups.

## Results

### Generation of multidrug-resistant leukemia K562 cell strains

#### Drug resistance and cross-resistance of K562

**Figure 1:**
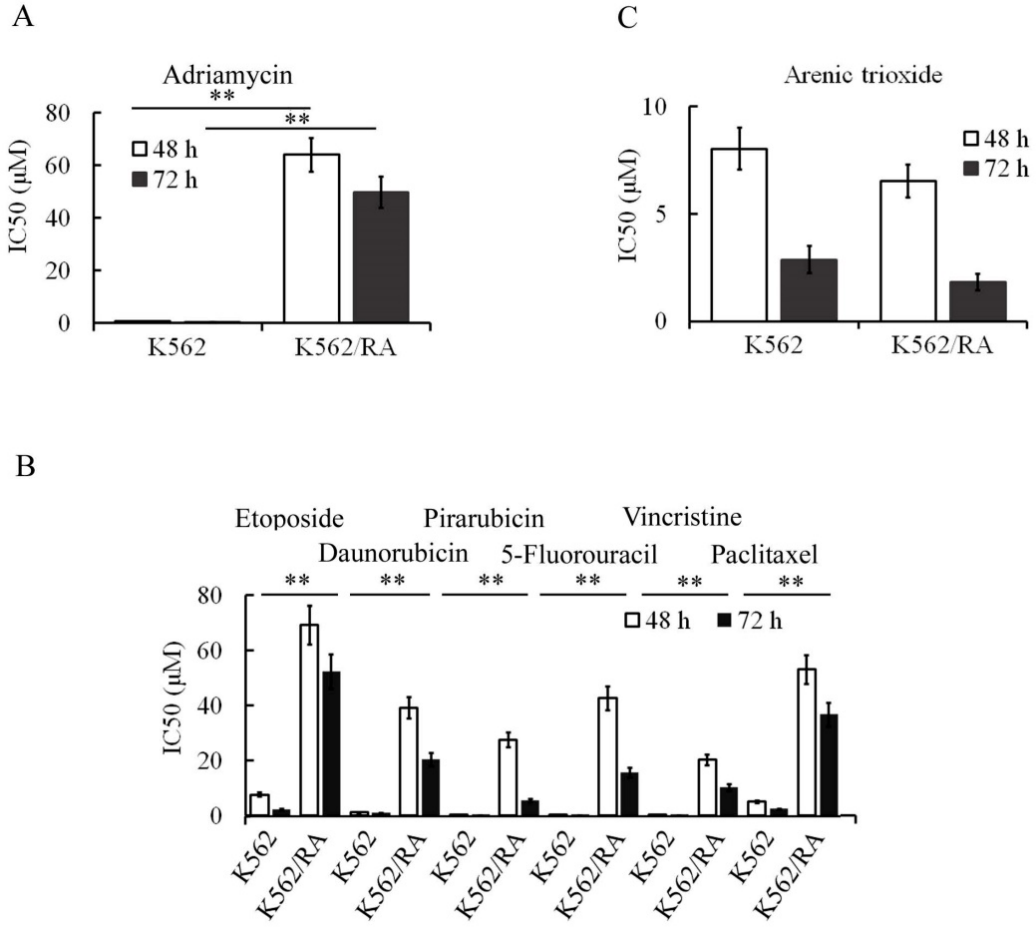
ADM promotes acquisition of multidrug resistance in K562 cells. After treatment, K562 cells developed resistance to ADM (A) in addition to other chemotherapeutic drugs, including pirarubicin, daunorubicin, vincristine, paclitaxel, etoposide and 5-FU (B). However, sensitivity of cells to ATO was not significantly changed (C). *P<0.05, **P<0.01, compared to K562 cells.

During the generation of ADM-induced resistance in K562 cells, tolerance of cells to ADM increased gradually with increasing concentrations and treatment durations. When cells could tolerate 16 µM ADM, IC_50_ values at 48 and 72 h for resistant cells were 84.2 and 94.2 times higher than that for parental cells, respectively (Figure 1A). Notably, these cells additionally developed resistance to other chemotherapeutic drugs including pirarubicin, daunorubicin, 5-FU, etoposide, vincristine and paclitaxel (Figure 1B), indicating that long-term and repeated stimulation with ADM induces acquisition of MDR in K562 cells. Interestingly, however, no significant cell resistance to ATO was evident (Figure 1C). K562 cells that tolerated 16 µM ADM were designated K562/RA.

#### Expression of drug-resistant genes and proteins in K562 cells during ADM induction of resistance

**Figure 2:**
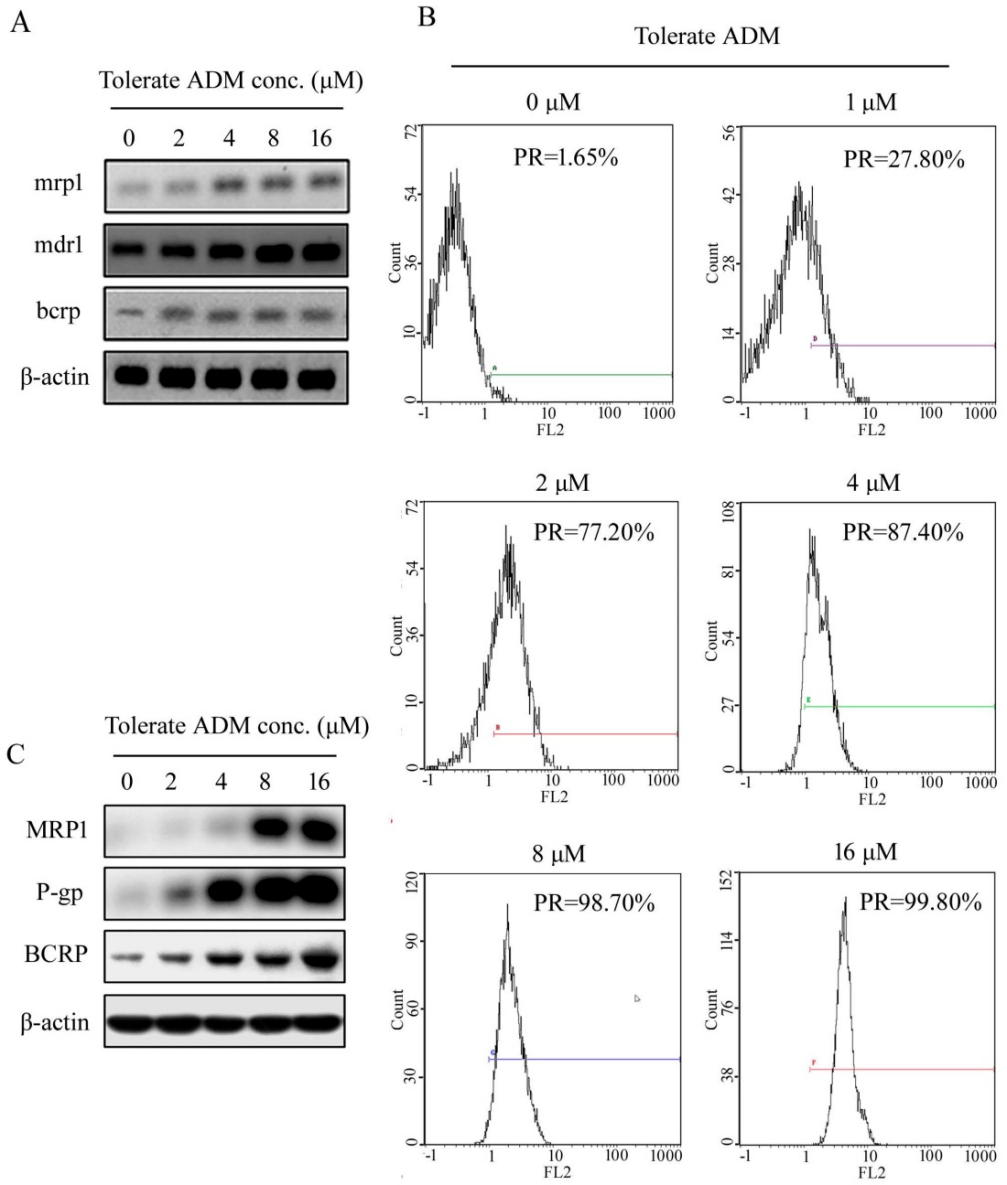
Expression of resistance-related genes and corresponding proteins in K562 cells tolerating different concentrations of ADM.Real-time RT-PCR analysis of relative expression of mdr1, mrp1 and bcrp mRNA in K562 cells with ADM resistance (A). Positive expression rate (PR) of P-gp detected with flow cytometry (B). Western blot analysis of intracellular P-gp, MRP1 and BCRP protein levels (C).

Initially, we observed extremely low expression of mdr1/P-gp, mrp1/MRP and bcrp/ BCRP in K562 cells. During the development of ADM-induced resistance of K562 cells, expression of mdr1, mrp1 and bcrp genes increased with the tolerable ADM concentration (Figure 2A). In K562 cells able to tolerate 16 µM ADM, expression of mdr1, mrp1 and brcp was 132.5-fold, 2.85-fold and 8.87-fold higher than that in parental cells, respectively. Flow cytometry (FCM) additionally showed an increase in the positive rate (PR) of P-gp protein expression and mean fluorescence indensity (MFI) with higher tolerable ADM concentrations. At a tolerable ADM concentration of 16 µM, nearly 100% cells displayed positive expression of P-gp (Figure 2B). Western blot data confirmed increased expression of P-gp, BCRP and MRP1 proteins in K562/RA cells. Notably, P-gp and MRP1 were almost undetectable in parental K562 but significantly upregulated in K562/RA cells (Figure 2C).

### Rsv enhanced the proliferation inhibitory activity of ATO on K562/RA cells

**Figure 3:**
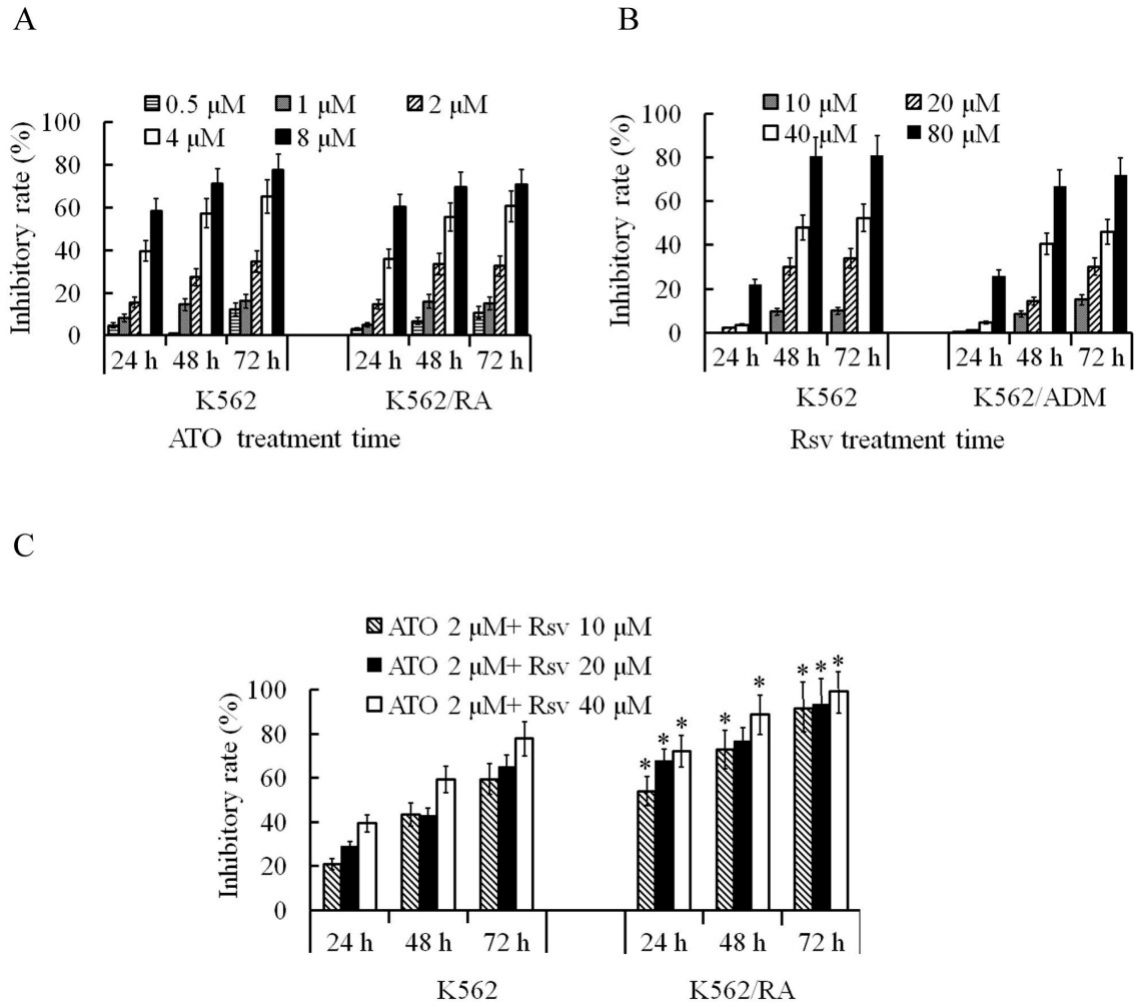
Inhibitory effects of ATO and Rsv on K562/RA cell proliferation. A, Comparison of the proliferation inhibitory rates of K562/RA and parental K562 cells after 24-72 h treatment with 0.5-8 μM ATO. B, Comparison of the proliferation inhibitory rates of K562/RA and parental K562 cells after 24-72 h treatment with 10-80 μM Rsv. C, Inhibitory effects of a combination of 2 μM ATO and 20 μM or 40 μmol/L Rsv on proliferative capacity. *P<0.05, compared to K562 cells.

After 24-72 h treatment with 0.5-8 μM ATO, proliferation rates of both K562/RA and parental K562 cells were significantly inhibited. The inhibitory rate was positively correlated with ATO concentration and treatment duration. Overall sensitivities of K562/RA and K562 cells to ATO were not significantly different (Figure 3A). After 24-72 h treatment with 10-80 μM Rsv, proliferation activity of K562/RA and K562 cells was also inhibited, with no significant differences in tolerance of the two cell types to Rsv (Figure 3B). Upon a combination treatment of 2 μM ATO and 20 or 40 μM Rsv (2 μM ATO + 20 μM Rsv and 2 μM ATO + 40 μM Rsv), the inhibitory rates in K562/RA cells were 67.7% and 72.1% after 24 h, 76.7% and 88.6% after 48 h and 93.4% and 99.1% at 72 h, respectively, compared to 28.8% and 39.4% after 24 h, 43% and 59.3% after 48 h and 65.2% and 77.7% at 72 h, respectively, in K562 cells. These data demonstrate that the efficacy of combination therapy with ATO and Rsv is significantly higher in K562/RA than parental K562 cells (Figure 3C), which suggests that Rsv enhances the suppressive effect of ATO in resistant cells.

### Rsv enhances the apoptosis-inducing effect of ATO in K562/RA cells

#### Morphological changes of apoptosis

**Figure 4:**
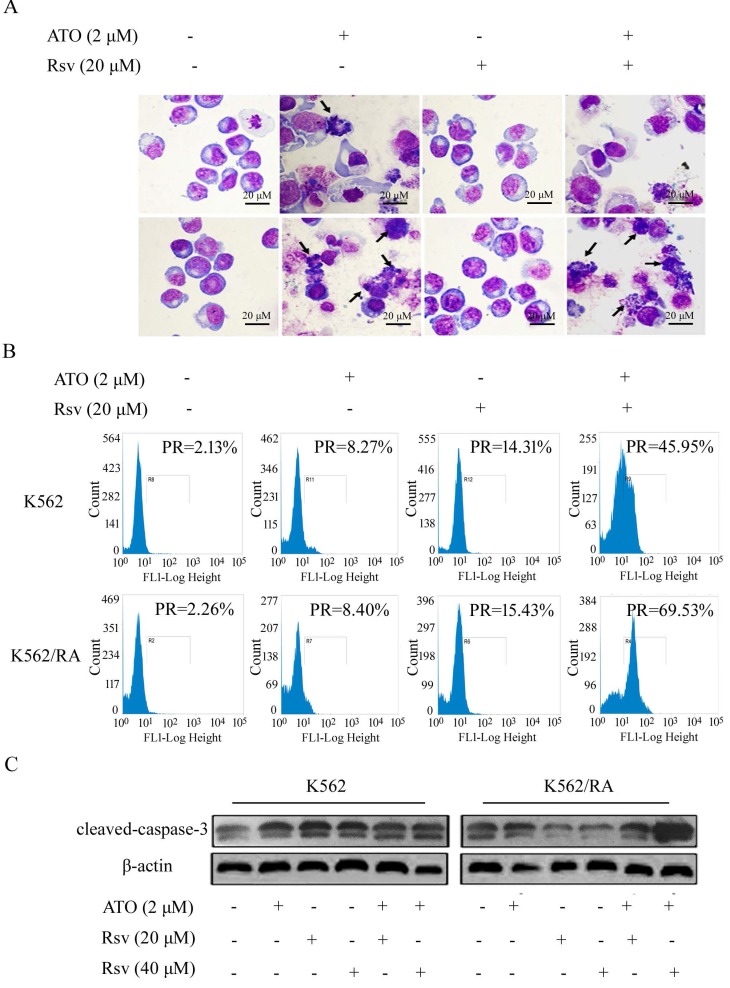
ATO combined with Rsv induces higher rates of K562/RA cell apoptosis. K562 or K562/RA cells were treated with ATO or Rsv alone or ATO combined with Rsv for 24 h. A, Morphological changes observed under inverted light microscopy (100×) via Swiss-Giemsa staining. The arrowhead (→) indicates chromatin condensation, nuclear fragmentation and apoptotic bodies in apoptotic cells; B, FCM analysis of the positive rate (PR), mean fluorescence indensity (MFI) and of activated intracellular caspase-3; C, Western blot detection of cleaved caspase-3 expression

We observed no significant morphological changes in K562 or K562/RA cells following treatment with 20 μM Rsv. After 24-h treatment with 2 μM ATO or 2 μM ATO plus 20 μM Rsv, morphological changes characteristic of apoptosis, such as chromatin condensation and cell shrinkage, were observed, which were more pronounced in cells treated with the drug combination (Figure 4A).

#### Caspase-3 activation

FCM analysis showed that after 24-h treatment with 2 μM ATO alone, the positive rate of caspase-3 activation in K562 and K562/RA groups increased from 2.13% and 2.26% in untreated control cells to 8.27% and 8.40%, respectively. After treatment with 40 μM Rsv alone, the positive rate increased to 22.19% and 16.78% while combined treatment with ATO and Rsv induced an increase in the rate of caspase-3 activation to 48.7% and 81.93%, respectively. Our results clearly indicate that both ATO and Rsv enhance caspase-3 activity in resistant and sensitive K562 cell types. Moreover, treatment with the ATO-Rsv combination promoted caspase-3 activity more significantly than either ATO or Rsv alone. This effect was more prominent in drug-resistant K562/RA cells (Figure 4B). The ATO and Rsv combination induced a significant increase in cleaved caspase-3 expression, particularly in K562/RA cells. These findings suggest that Rsv significantly enhances the apoptosis-inducing effect of ATO in drug-resistant cells (Figure 4C).

#### BAX/BCL-2 expression

**Figure 5:**
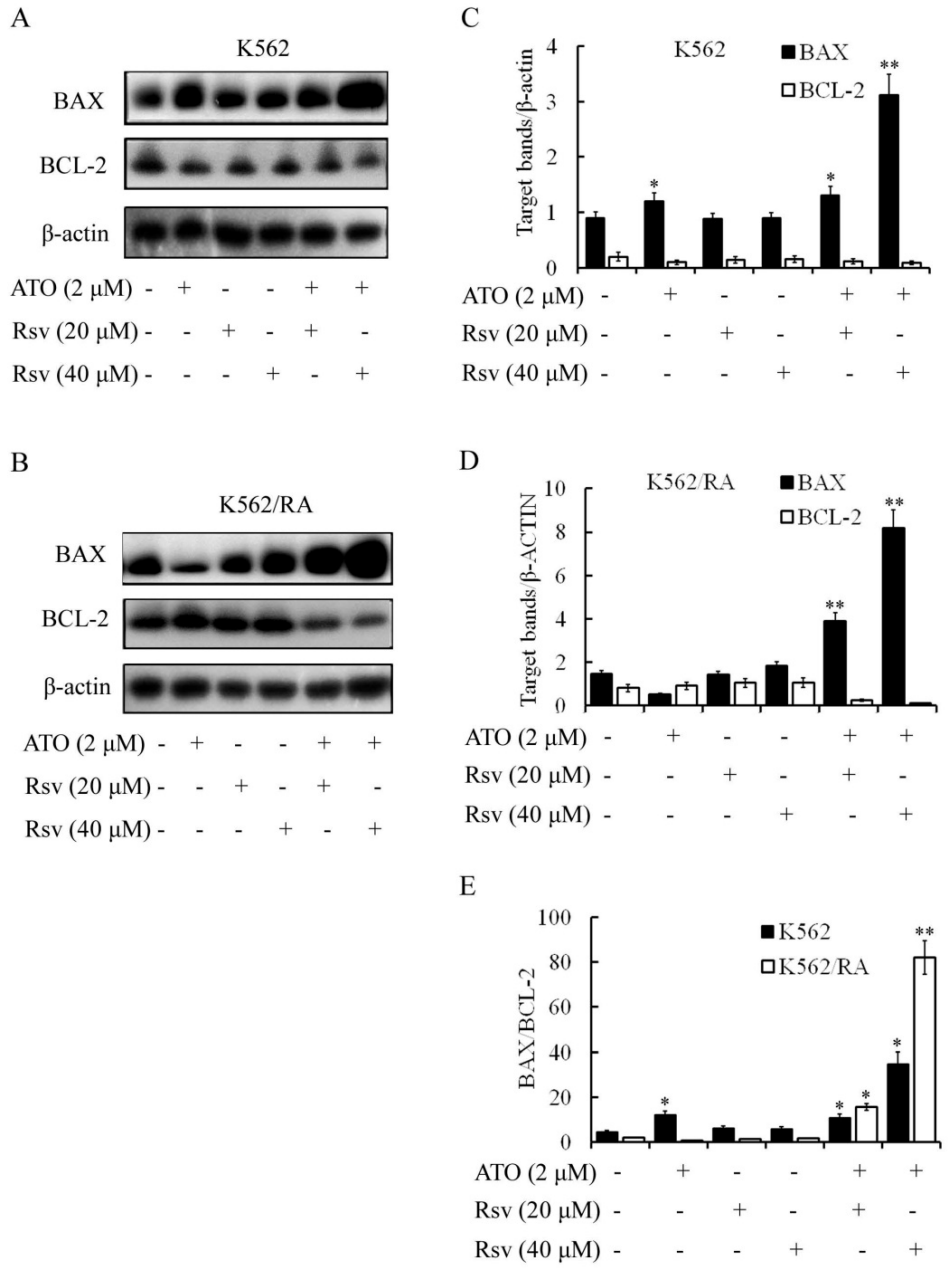
Effects of combination treatment with ATO and Rsv on BAX/BCL-2 expression.BAX and BCL-2 protein levels in K562 (A) and K562/RA (B) cells were detected via western blot after 24-h treatment with ATO or Rsv alone or a combination of ATO and Rsv. C, D, Corresponding relative protein levels of antibody-coated protein bands in immunoblots (scaned using an Odyssey double-color infrared-laser imaging system and their OD values were analyzed using an Odyssey v1.2 software. The relative protein levels were calculated by comparison to that of β-actin via the OD_interest protein_ / OD_β-actin protein_ method). E, Comparison of the BAX/BCL-2 ratio between the two cell types. *P<0.05, **P<0.01, compared to control.

Treatment with 2 μM ATO or 20 or 40 μM Rsv alone led to significant upregulation of BAX protein in K562 and K562/RA cells. Concomitantly, expression of BCL-2 was weakly downregulated. The BAX/BCL-2 ratios in K562 cells treated with 2 μM ATO, 20 μM Rsv and 40 μM Rsv were 2.67-, 1.39- and 1.32-fold higher than that of the control group, respectively (Figure 5A-D). After combination treatment with 2 μM ATO and 20 μM or 40 μM Rsv, the BAX/BCL-2 ratio was 2.42- and 7.7-fold higher than that of the control group for the K562 cell line. For the K562/RA cell line, the ratio was 20.27- and 97.33-fold higher in the treatment groups, respectively (Figure 5E). Our results suggest that Rsv enhances the apoptosis-inducing effect of ATO via concomitantly upregulating BAX and downregulating BCL-2. This synergistic apoptosis-promoting activity is more pronounced in drug-resistant K562/RA cells.

#### P53 expression

**Figure 6:**
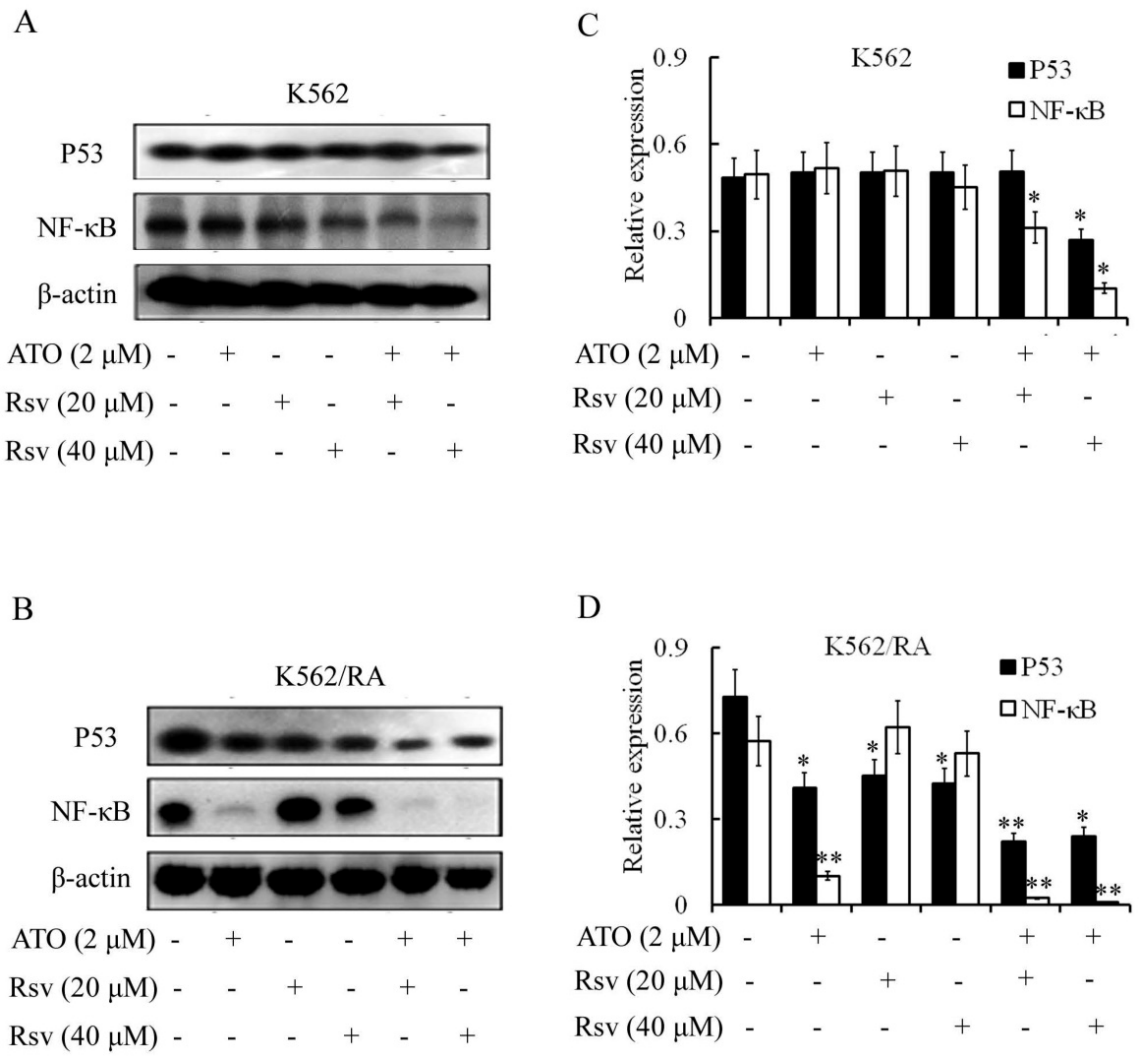
Combination treatment with ATO and Rsv inhibits P53 and NF-*κ*B expression. After 24-h treatment with ATO or Rsv alone or ATO plus Rsv, western blot was applied to detect expression of P53 and NF-κB proteins in K562 (A) and K562/RA cells (B). C, D, Corresponding OD values. *P<0.05, **P<0.01, compared to control.

Treatment with 2 µM ATO, 20 µM or 40 µM Rsv alone had no significant effects on P53 levels. However, expression of P53 was markedly decreased in K562 and K562/RA cells after combined treatment with ATO and Rsv (Figure 6A-D). In particular, co-treatment with 2 µM ATO and 20 µM or 40 µM Rsv induced a more significant decrease in P53 expression in K562/RA cells (Figure 6A-D).

#### NF-κB expression

Activated NF-κB inhibits apoptosis. Treatment with 2 µM ATO led to significant downregulation of NF-κB expression in K562/RA but exerted a weak effect on K562 cells (Figure 6A and B). Expression of NF-κB was not affected upon treatment with 20 or 40 µM Rsv. However, 2 µM ATO combined with 20 or 40 µM Rsv induced downregulation of NF-κB in both K562 and K562/RA cell lines. In K562 cells, the combination treatment suppressed NF-κB expression to 63.03% and 20.61% relative to the control group, while in K562/RA cells, expression decreased to only 4.02% and 1.75% that of the control group (Figure 6C and D), suggesting that ATO and Rsv act synergistically to induce apoptosis in cells by inhibiting the NF-*κ*B signaling pathway, particularly in drug-resistant leukemia cells.

### Rsv enhances the inhibitory effect of ATO on expression of resistance-related genes in K562/RA cells

**Figure 7:**
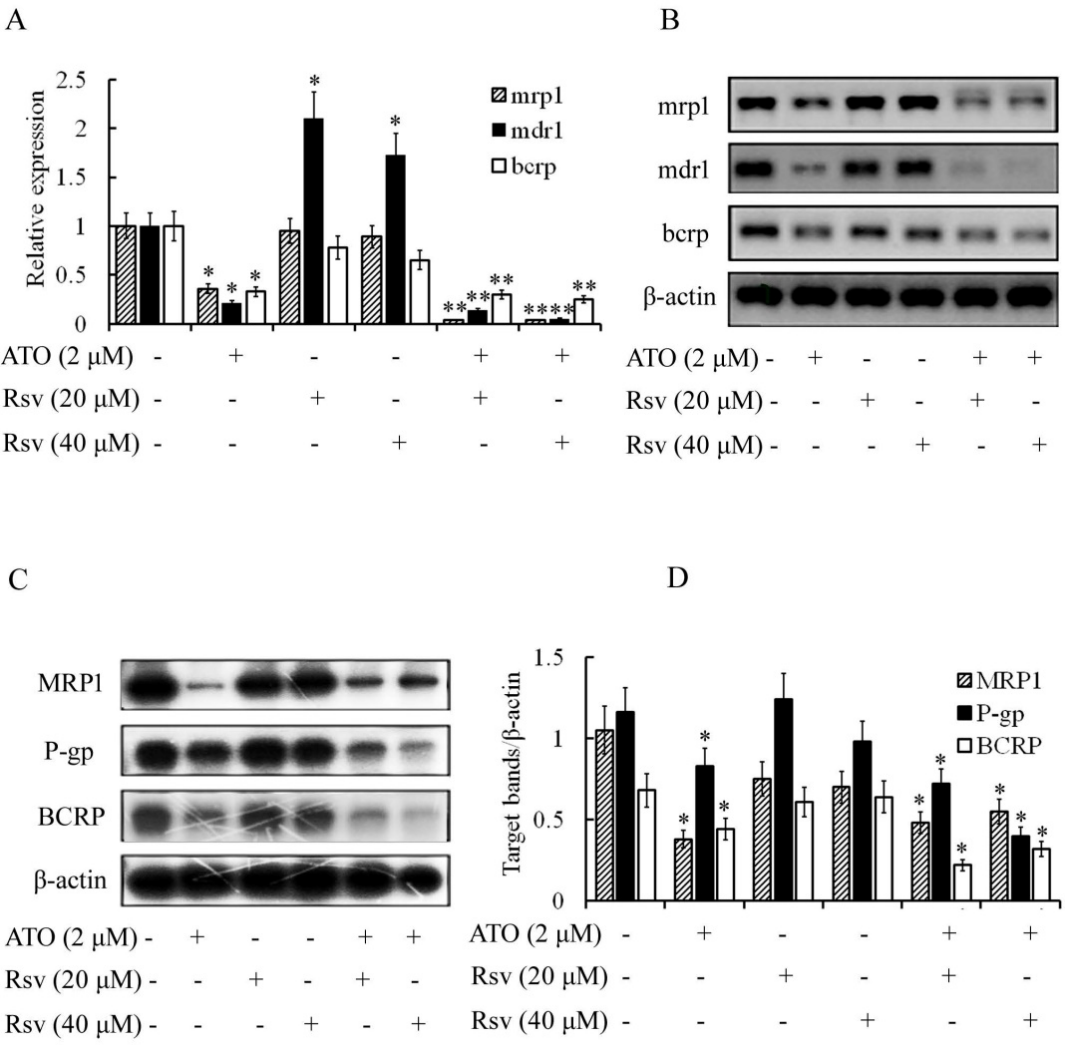
ATO and Rsv synergistically inhibit resistance-related genes. After 24-h treatment with ATO or Rsv alone or the combination of ATO and Rsv, real-time RT-PCR was used to detect expression of mdr1, mrp1 and bcrp genes in K562 and K562/RA cells (A) and the corresponding electrophoresis spectrum of the amplified products (B). Western blot detection of expression of P-gp, MRP1 and BCRP proteins (C) and the corresponding OD values (D). *P<0.05, **P<0.01, compared to control.

Expression of mdr1, bcrp and mrp1 was high in ADM-induced resistant K562/RA cells but nearly undetectable in parental K562 cells (Figure 2). ATO (2 μM) significantly inhibited expression of these genes in K562/RA cells (21%, 33% and 36%, compared to the control group, respectively). Rsv alone (20 μM or 40 μM) had no or a weak inhibitory effect on the genes responsible for drug resistance, while combined treatment with 2 μM ATO and 20 or 40 μM Rsv synergistically downregulated the expression of drug-resistant genes in K562/RA cells. Levels of mdr1 were only 15% and 5% while those of bcrp were 30% and 25% relative to the control group, respectively, and 0.4% for mrp1, as detected with real time RT-PCR (Figure 7A and B). Western blot results were in line with RT-PCR findings (Figure 7C and D), further confirming that ATO combined with Rsv exerts a stronger apoptotic effect on K562/RA cells. The underlying mechanisms may be associated with downregulation of resistance-related genes through synergistic effects of ATO and Rsv.

## Discussion

ATO has been widely used in the management of leukemia and shown to be effective in treating a variety of solid tumors, including lung, liver, ovarian, colon and esophageal cancer types 4,7-12. The mechanisms underlying the anti-tumor activity of ATO are complex. ATO can induce tumor cell differentiation, promote degradation of PML/RARα fusion protein, block specific intracellular signaling pathways, and trigger apoptosis by activating the intracellular caspase cascade, downregulating Bcl-2 expression and inhibiting NF-kB. The apoptosis-inducing effect of ATO on leukemia and cancer cells represents the key mechanism that contributes to its anti-tumor activity 4,7-12,24. In 1998, Rsv was initially reported to induce apoptosis of human leukemia HL-60 cells via the Fas-FasL pathway with no apparent side-effects on peripheral lymphocytes 25,26. Subsequent studies confirmed that Rsv exerts apoptotic effects on a variety of tumor cells, including T47D human breast cancer, U87 glioma, K562 leukemia, lymphoma and prostate cancer cells, with no significant side-effects on normal cells 22,25-29. Notably, the compound has a strong killing effect on cancer stem cells. Additionally, previous experiments by our group have confirmed that Rsv induces apoptosis in leukemia K562 and stem cells.

Recent studies suggest that Rsv not only has a synergistic inhibitory effect with ATO on tumor cells, but also reduces the toxicity of ATO alone. Here, we combined a lower concentration of ATO with Rsv to determine the proliferation inhibitory and apoptosis-inducing effects on resistant K562/RAcells. ATO, in combination with Rsv, exerted a synergistic inhibitory effect on proliferation of both K562/RA and parental K562 cells, which was more pronounced in the resistant cell line. The cells underwent morphological changes typical of apoptosis, and the apoptotic rate was significantly increased. Although caspase-3 expression was not affected to a marked extent, its activity was remarkably enhanced, particularly in K562/RA cells, indicating that Rsv enhances the proliferation inhibitory and apoptotic effects of ATO on K562/RA cells.

Interactions between BCL-2 on the mitochondrial membrane and BAX in the cytoplasm affect the stability of the mitochondrial membrane and determine cell apoptosis 28. ATO combined with Rsv suppressed the expression of BCL-2 and P53 and increased the expression of BAX, thereby increasing the BAX/BCL-2 ratio. In drug-resistant K562/RA cells, Rsv and ATO exerted a stronger combined effect on downregulation of P53, which could trigger changes in the expression of downstream genes, such as BCL-2 and Bax. Accordingly, the permeability of the mitochondrial membrane was increased and CytC released, further activating caspase cascade reactions, leading to cellular apoptosis. NF-*κ*B is a transcription factor and the associated signaling pathway is inactive in normal cells but continuously activated in tumor cells. NF-κB activation leads to upregulation of the anti-apoptotic protein, BCL-2, which downregulates IκBα expression to further enhance activation of NF-*κ*B via a feedback mechanism. The transcription factor plays a critical role in the proliferation and apoptosis of tumor cells. We propose that ATO and Rsv synergistically inhibit the expression and activation of NF-κB in K562/RA cells, thus suppressing its promotory effect on BCL-2 expression to induce cell apoptosis.

The membrane transporters P-gp, MRP1 and BCRP protect cells against injury induced by chemotherapeutic drugs and other exotoxins. However, these proteins also mediate the development of multidrug resistance in tumor cells. Meanwhile, evidence has shown that P-gp participates in the regulation of cellular apoptosis. In our experiments, the levels of mdr1, bcrp and mrp1 genes in K562/RA cells were significantly higher than those of parental K562 cells. Rsv did not affect expression of mdr1, bcrp and mrp1 in K562/RA cells but significantly enhanced the inhibitory effects of ATO on gene expression. These results suggest that Rsv and ATO synergistically enhance apoptosis of K562/RA cells, which may be associated with inhibition of mdr1, bcrp and mrp1. Additional studies 30 have shown that the promoter of mdr1 contains the binding site of NF-κB. Binding of NF-κB activates the transcription of mdr1 and upregulates mdr1/P-gp. In tumor cells, mutated P53 can also activate the mdr1 promoter and upregulate the gene at the transcriptional level. Moreover, overexpression of BCL-2 promotes mdr1 expression. The collective findings support a complex regulatory network constituting apoptosis-related and drug-resistant genes that act in concert to modulate drug resistance and apoptosis of resistant leukemia cells.

## Conclusions

Rsv enhances the apoptosis-inducing effect of ATO on both drug-resistant K562/RA and parental K562 cells and significantly suppresses expression of the resistance-related genes mdr1/P-gp, mrp1/MRP1 and bcrp/BCRP. Moreover, apoptosis induced by Rsv and ATO is associated with the Bcl-2/Bax and NF-κB pathways. Further research is warranted to elucidate the detailed mechanisms underlying the synergistic apoptotic effects of these drugs.
